# Supplementary material for: Diversity in the intrinsic apoptosis pathway of nematodes
Source: Commun Biol. 2020 Aug 28;3:478. doi: 10.1038/s42003-020-01208-5 (PMC7456325; doi:10.1038/s42003-020-01208-5)
Supplement: Supplementary file 1 — Supplementary Materials [file 42003_2020_1208_MOESM1_ESM.pdf]

Supplementary Material

Diversity in the Intrinsic Apoptosis Pathway of Nematodes

Neil D. Young, Tiffany J. Harris, Marco Evangelista, Sharon Tran, Merridee Wouters, Tatiana P. Soares da Costa, Nadia J. Kershaw, Robin B. Gasser, Brian J. Smith, Erinna F. Lee, W. Douglas Fairlie

This file includes the following Supplementary Material:

Supplementary Figure 1
Supplementary Figure 2
Supplementary Figure 3
Supplementary Figure 4
Supplementary Figure 5
Supplementary Table 1
Supplementary Table 2
Supplementary Table 3
Supplementary Table 4
Supplementary Table 5
Supplementary Table 6

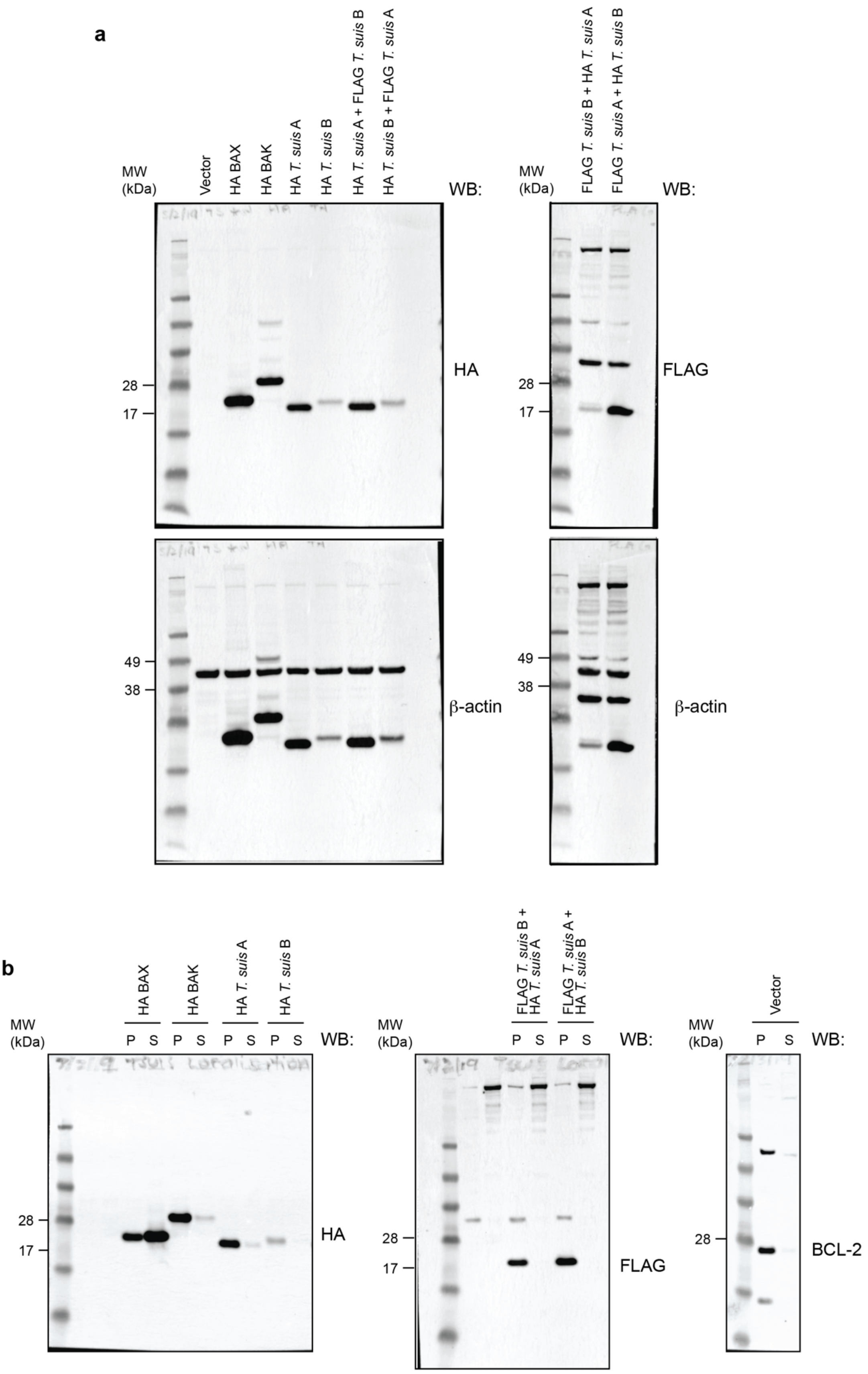

Supplementary Figure 1: Uncropped blots used to construct a) Figure 5a and b) Figure 5b.

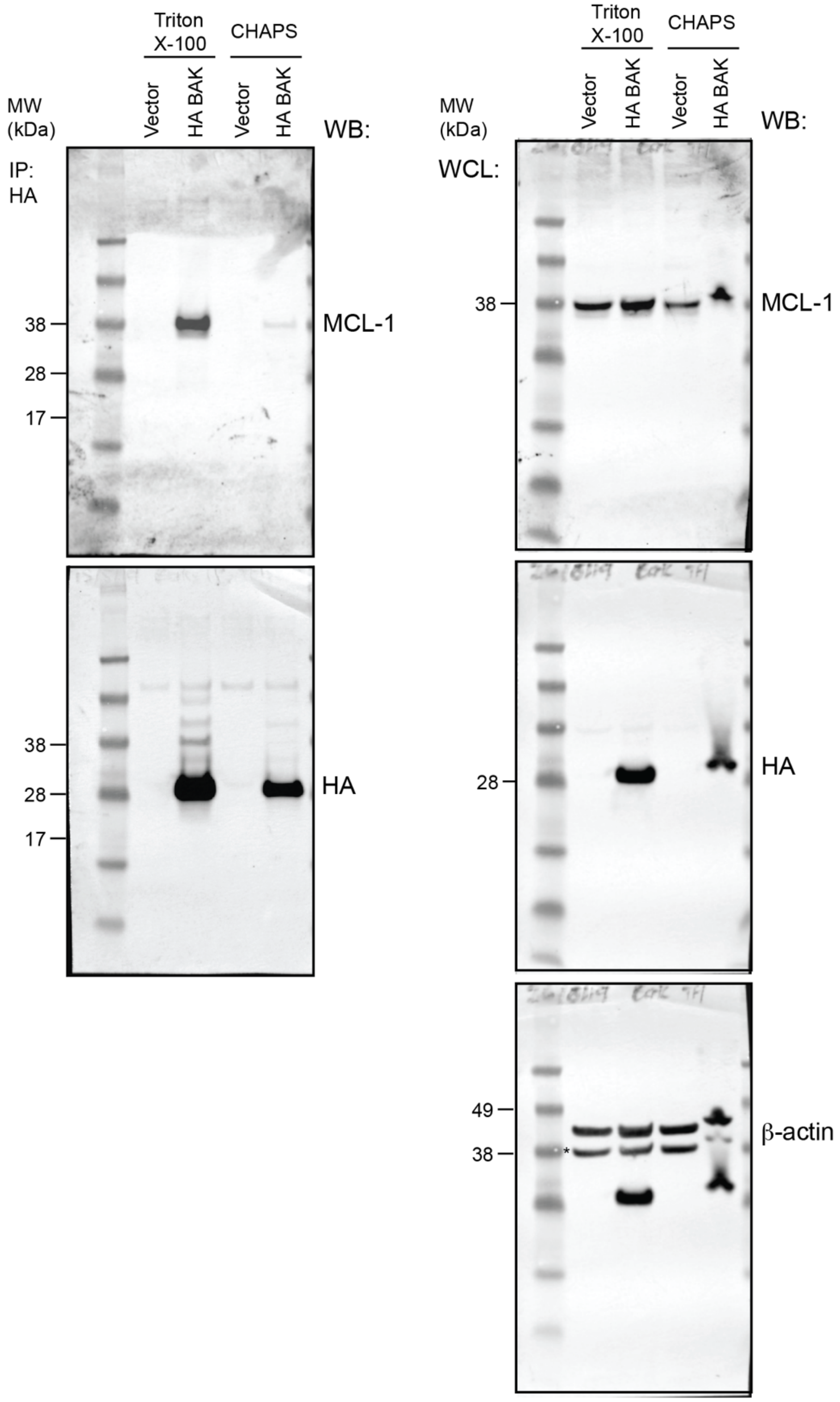

Supplementary Figure 2: Uncropped blots used to construct Figure 5c (Part 1) – MCL-1 + BAK immunoprecipitation.

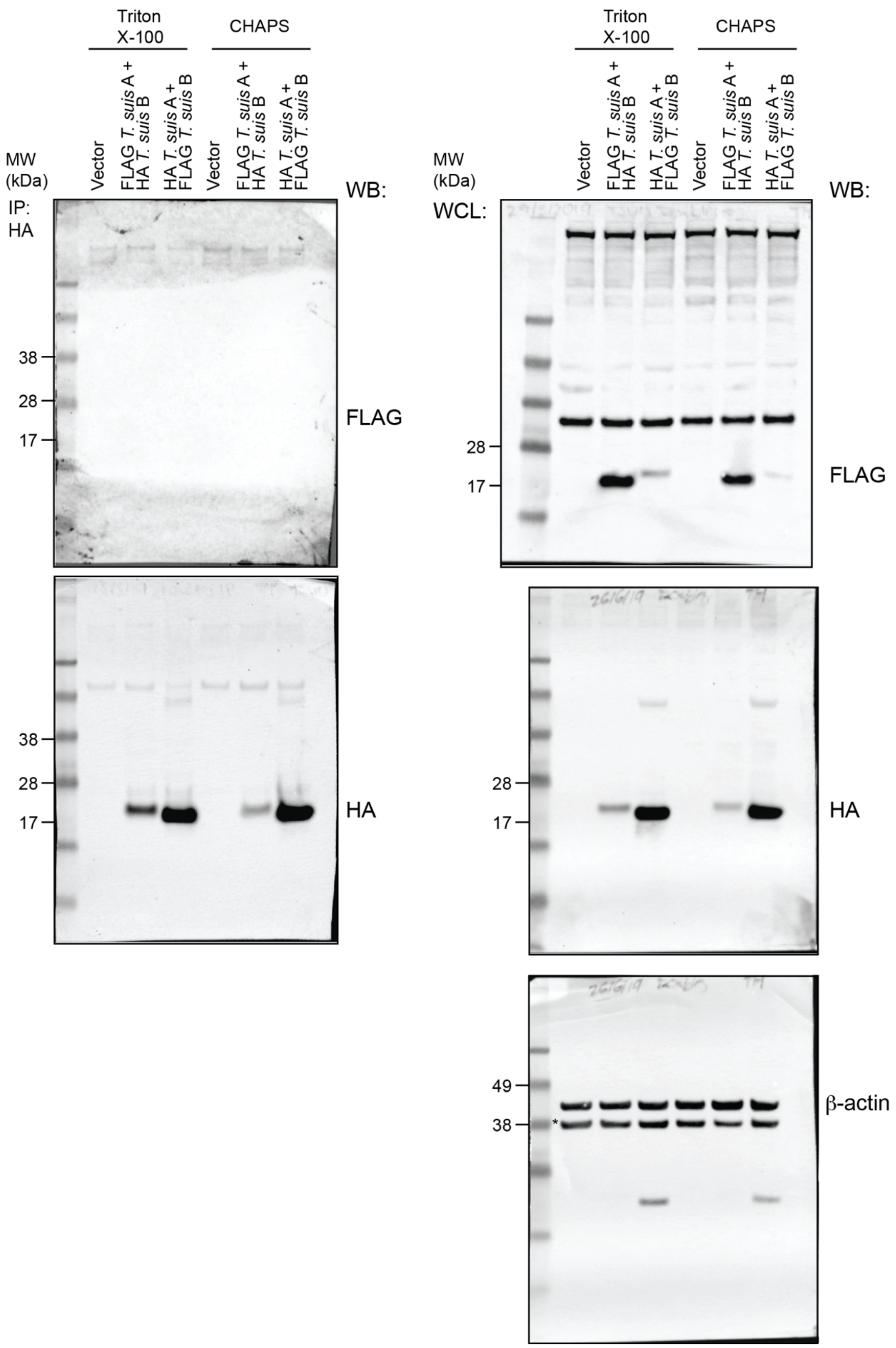

Supplementary Figure 3: Uncropped blots used to construct Figure 5c (Part 2) – *T.suis* A + B immunoprecipitation.

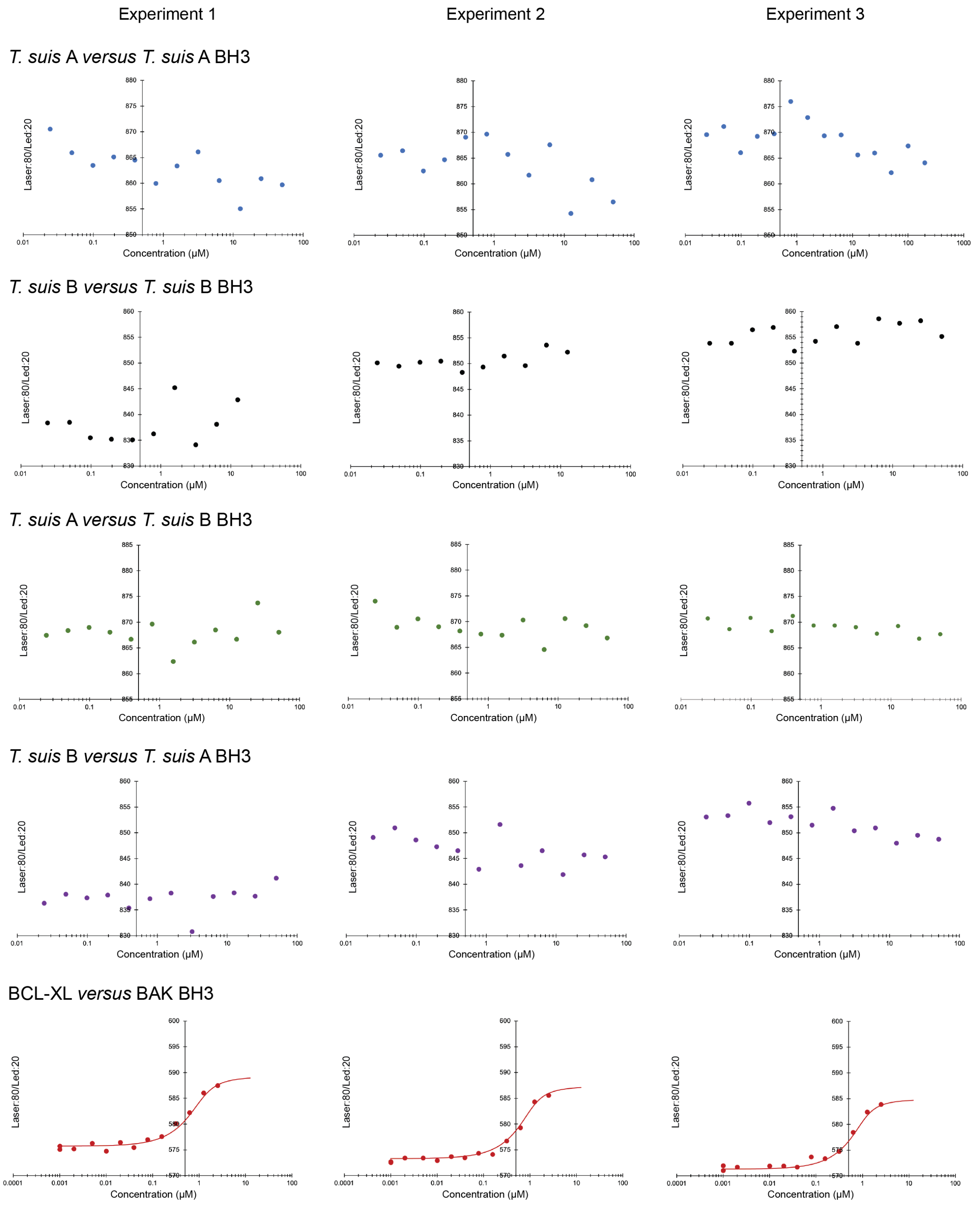

Supplementary Figure 4: Microscale thermophoresis analysis of interactions between *T. suis* A and *T. suis* B with synthetic peptides corresponding to their own or the other's BH3 domain. No binding was observed with any of these interactions whilst the positive control experiments showed an obvious interaction between BCL-XL and a BAK BH3 domain synthetic peptide.

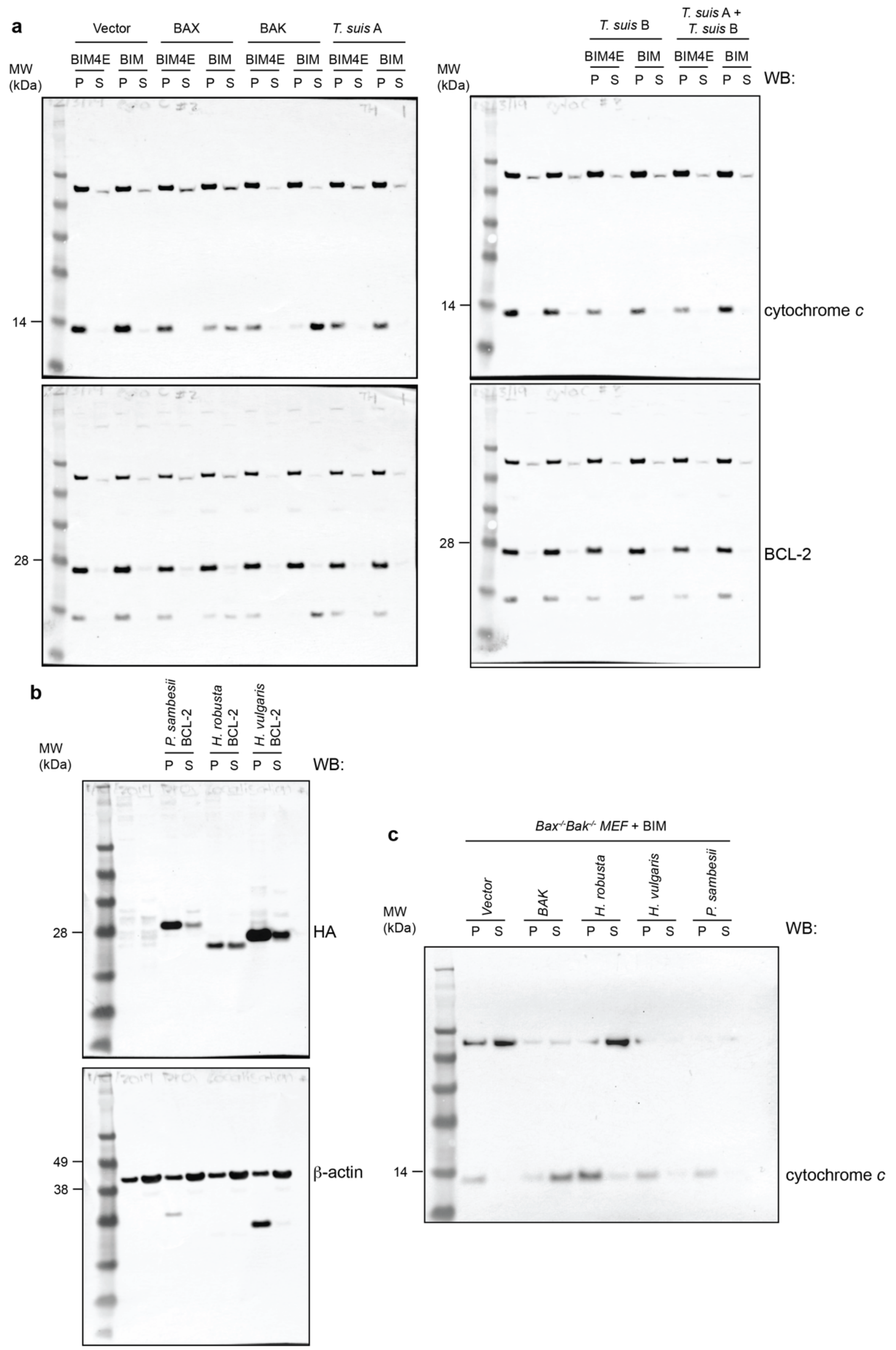

Supplementary Figure 5: Uncropped blots used to construct a) Figure 6b, b) Figure 6c, c) Figure 6d.

Supplementary Table 1: Summary of the species selected for analysis selected from the WormBase:Parasite and ENSEMBL Metazoa websites.

Species	Genome identifier in database	Group	Nematode clade	Free-living/Parasites definitive host	Database
Plectus sambesii	plectus_sambesii_prjna390260	Nematoda	C	Free-living	WormBase
Romanomermis culicivorax	romanomermis_culicivorax_prjeb1358	Nematoda	I	Parasitic: Invertebrate	WormBase
Soboliphyme baturini	soboliphyme_baturini_prjeb516	Nematoda	I	Parasitic: Vertebrate	WormBase
Trichinella britovi	trichinella_britovi_prjna257433	Nematoda	I	Parasitic: Vertebrate	WormBase
Trichinella murrelli	trichinella_murrelli_prjna257433	Nematoda	I	Parasitic: Vertebrate	WormBase
Trichinella nativa	trichinella_nativa_prjna179527	Nematoda	I	Parasitic: Vertebrate	WormBase
Trichinella nelsoni	trichinella_nelsoni_prjna257433	Nematoda	I	Parasitic: Vertebrate	WormBase
Trichinella papuae	trichinella_papuae_prjna257433	Nematoda	I	Parasitic: Vertebrate	WormBase
Trichinella patagoniensis	trichinella_patagoniensis_prjna257433	Nematoda	I	Parasitic: Vertebrate	WormBase
Trichinella spiralis	trichinella_spiralis_prjna12603; trichinella_spiralis_prjna257433; Trichinella_spiralis.Tspiralis1.pep.all.fa	Nematoda	I	Parasitic: Vertebrate	WormBase; ENSEMBL Metazoa
Trichinella t6	trichinella_t6_prjna257433	Nematoda	I	Parasitic: Vertebrate	WormBase
Trichinella t8	trichinella_t8_prjna257433	Nematoda	I	Parasitic: Vertebrate	WormBase
Trichinella t9	trichinella_t9_prjna257433	Nematoda	I	Parasitic: Vertebrate	WormBase
Trichinella zimbabwensis	trichinella_zimbabwensis_prjna257433	Nematoda	I	Parasitic: Vertebrate	WormBase
Trichuris muris	trichuris_muris_prjeb126	Nematoda	I	Parasitic: Vertebrate	WormBase
Trichuris suis	trichuris_suis_prjna179528;trichuris_suis_prjna208415:trichuris_suis_prjna208416	Nematoda	I	Parasitic: Vertebrate	WormBase
Trichuris trichiura	trichuris_trichiura_prjeb535	Nematoda	I	Parasitic: Vertebrate	WormBase
Xiphinema index	Not applicable	Nematoda	I	Parasitic: Plant	Furlanetto et al, (2005) Nematology 7: 95-104.
Acanthocheilonema viteae	acanthocheilonema_viteae_prjeb4306	Nematoda	III	Parasitic: Vertebrate	WormBase
Anisakis simplex	anisakis_simplex_prjeb496	Nematoda	III	Parasitic: Vertebrate	WormBase
Ascaris lumbricoides	ascaris_lumbricoides_prjeb4950	Nematoda	III	Parasitic: Vertebrate	WormBase
Ascaris suum	ascaris_suum_prjna62057; ascaris_suum_prjna80881	Nematoda	III	Parasitic: Vertebrate	WormBase
Brugia malayi	brugia_malayi_prjna10729; Brugia_malayi.Bmal-4.0.pep.all.fa	Nematoda	III	Parasitic: Vertebrate	WormBase; ENSEMBL Metazoa
Brugia pahangi	brugia_pahangi_prjeb497	Nematoda	III	Parasitic: Vertebrate	WormBase
Brugia timori	brugia_timori_prjeb4663	Nematoda	III	Parasitic: Vertebrate	WormBase
Dirofilaria immitis	dirofilaria_immitis_prjeb1797	Nematoda	III	Parasitic: Vertebrate	WormBase
Dracunculus medinensis	dracunculus_medinensis_prjeb500	Nematoda	III	Parasitic: Vertebrate	WormBase
Elaeophora elaphi	elaeophora_elaphi_prjeb502	Nematoda	III	Parasitic: Vertebrate	WormBase
Enterobius vermicularis	enterobius_vermicularis_prjeb503	Nematoda	III	Parasitic: Vertebrate	WormBase
Litomosoides sigmodontis	litomosoides_sigmodontis_prjeb3075	Nematoda	III	Parasitic: Vertebrate	WormBase
Loa loa	loa_loa_prjna246086; loa_loa_prjna60051;Loa_loa.Loa_loa_V3.pep.all.fa	Nematoda	III	Parasitic: Vertebrate	WormBase; ENSEMBL Metazoa
Onchocerca flexuosa	onchocerca_flexuosa_prjeb512	Nematoda	III	Parasitic: Vertebrate	WormBase
Onchocerca ochengi	onchocerca_ochengi_prjeb1204; onchocerca_ochengi_prjeb1809	Nematoda	III	Parasitic: Vertebrate	WormBase
Onchocerca volvulus	onchocerca_volvulus_prjeb513; Onchocerca_volvulus.ASM49940v2.pep.all.fa	Nematoda	III	Parasitic: Vertebrate	WormBase; ENSEMBL Metazoa
Parascaris univalens	parascaris_univalens_prjna386823	Nematoda	III	Parasitic: Vertebrate	WormBase
Syphacia muris	syphacia_muris_prjeb524	Nematoda	III	Parasitic: Vertebrate	WormBase
Thelazia callipaeda	thelazia_callipaeda_prjeb1205	Nematoda	III	Parasitic: Vertebrate	WormBase
Toxocara canis	toxocara_canis_prjeb533; toxocara_canis_prjna248777	Nematoda	III	Parasitic: Vertebrate	WormBase
Wuchereria bancrofti	wuchereria_bancrofti_prjna275548	Nematoda	III	Parasitic: Vertebrate	WormBase
Acrobeloides nanus	acrobeloides_nanus_prjeb26554	Nematoda	IV	Free-living	WormBase
Globodera rostochiensis	globodera_rostochiensis.PRJEB13504.WBPS12	Nematoda	IV	Parasitic: Plant	WormBase
Meloidogyne arenaria	meloidogyne_arenaria.PRJEB8714.WBPS12	Nematoda	IV	Parasitic: Plant	WormBase
Meloidogyne floridensis	meloidogyne_floridensis.PRJEB6016.WBPS12	Nematoda	IV	Parasitic: Plant	WormBase
Meloidogyne graminicola	meloidogyne_graminicola.PRJNA411966.WBPS12	Nematoda	IV	Parasitic: Plant	WormBase
Meloidogyne javanica	meloidogyne_javanica.PRJEB8714.WBPS12	Nematoda	IV	Parasitic: Plant	WormBase
Steinernema carpocapsae	steinernema_carpocapsae_prjna202318	Nematoda	IV	Parasitic: Invertebrate	WormBase
Steinernema feltiae	steinernema_feltiae_prjna204661	Nematoda	IV	Parasitic: Invertebrate	WormBase
Steinernema glaseri	steinernema_glaseri_prjna204943	Nematoda	IV	Parasitic: Invertebrate	WormBase
Steinernema scapterisci	steinernema_scapterisci_prjna204942	Nematoda	IV	Parasitic: Invertebrate	WormBase
Strongyloides ratti	Strongyloides_ratti.S_ratti_ED321_v5_0_4.pep.all.fa	Nematoda	IV	Parasitic: Vertebrate	ENSEMBL Metazoa
Ancylostoma caninum	ancylostoma_caninum_prjna72585	Nematoda	V	Parasitic: Vertebrate	WormBase
Ancylostoma ceylanicum	ancylostoma_ceylanicum_prjna231479	Nematoda	V	Parasitic: Vertebrate	WormBase
Ancylostoma duodenale	ancylostoma_duodenale_prjna72581	Nematoda	V	Parasitic: Vertebrate	WormBase
Angiostrongylus cantonensis	angiostrongylus_cantonensis_prjeb493	Nematoda	V	Parasitic: Vertebrate	WormBase
Angiostrongylus costaricensis	angiostrongylus_costaricensis_prjeb494	Nematoda	V	Parasitic: Vertebrate	WormBase
Caenorhabditis angaria	caenorhabditis_angaria_prjna51225	Nematoda	V	Free-living	WormBase
Caenorhabditis brenneri	caenorhabditis_brenneri_prjna20035;Caenorhabditis_brenneri.C_brenneri-6.0.1b.pep.all.fa	Nematoda	V	Free-living	WormBase; ENSEMBL Metazoa
Caenorhabditis briggsae	caenorhabditis_briggsae_prjna10731;Caenorhabditis_briggsae.CB4.pep.all.fa	Nematoda	V	Free-living	WormBase; ENSEMBL Metazoa
Caenorhabditis elegans	caenorhabditis_elegans_prjna13758; Caenorhabditis_elegans.WBcel235.pep.all.fa	Nematoda	V	Free-living	WormBase; ENSEMBL Metazoa
Caenorhabditis sp.	Caenorhabditis sp.	Nematoda	V	Free-living	NCBI
Caenorhabditis japonica	Caenorhabditis_japonica.C_japonica-7.0.1.pep.all.fa	Nematoda	V	Free-living	ENSEMBL Metazoa
Caenorhabditis latens	caenorhabditis_latens_prjna248912	Nematoda	V	Free-living	WormBase
Caenorhabditis nigoni	caenorhabditis_nigoni_prjna384657	Nematoda	V	Free-living	WormBase
Caenorhabditis remanei	caenorhabditis_remanei_prjna248909, prjna248911, prjna53967,C_remanei-15.0.1.pep.all.fa	Nematoda	V	Free-living	WormBase; ENSEMBL Metazoa
Caenorhabditis sinica	caenorhabditis_sinica_prjna194557	Nematoda	V	Free-living	WormBase
Caenorhabditis sp34	caenorhabditis_sp34_prjdb5687	Nematoda	V	Free-living	WormBase
Caenorhabditis tropicalis	caenorhabditis_tropicalis_prjna53597	Nematoda	V	Free-living	WormBase
Dictyocaulus filaria	Dictyocaulus filaria	Nematoda	V	Parasitic: Vertebrate	Mangiola, S. et al., (2013) Biotechnol Adv 31: 1109-1119
Dictyocaulus viviparus	dictyocaulus_viviparus_prjeb5116; dictyocaulus_viviparus_prjna72587	Nematoda	V	Parasitic: Vertebrate	WormBase
Diploscapter coronatus	diploscapter_coronatus_prjdb3143	Nematoda	V	Free-living	WormBase
Diploscapter pachys	diploscapter_pachys_prjna280107	Nematoda	V	Free-living	WormBase
Haemonchus contortus	haemonchus_contortus_prjeb506; haemonchus_contortus_prjna205202	Nematoda	V	Parasitic: Vertebrate	WormBase
Haemonchus placei	haemonchus_placei_prjeb509	Nematoda	V	Parasitic: Vertebrate	WormBase
Heligmosomoides polygyrus	heligmosomoides_polygyrus_prjeb1203; heligmosomoides_polygyrus_prjeb15396	Nematoda	V	Parasitic: Vertebrate	WormBase
Nippostrongylus brasiliensis	nippostrongylus_brasiliensis_prjeb511	Nematoda	V	Parasitic: Vertebrate	WormBase
Oesophagostomum dentatum	oesophagostomum_dentatum_prjna72579	Nematoda	V	Parasitic: Vertebrate	WormBase
Oscheius tipulae	oscheius_tipulae_prjeb15512	Nematoda	V	Free-living	WormBase
Pristionchus exspectatus	pristionchus_exspectatus_prjeb6009	Nematoda	V	Free-living	WormBase
Pristionchus pacificus	pristionchus_pacificus_prjna12644; Pristionchus_pacificus.P_pacificus-5.0.pep.all.fa	Nematoda	V	Free-living	WormBase; ENSEMBL Metazoa
Strongylus vulgaris	strongylus_vulgaris_prjeb531	Nematoda	V	Parasitic: Vertebrate	WormBase
Teladorsagia circumcincta	teladorsagia_circumcincta_prjna72569	Nematoda	V	Parasitic: Vertebrate	WormBase
Trichostrongylus colubriformis	T_colubriformis	Nematoda	V	Parasitic: Vertebrate	Mangiola, S. et al., (2013) Biotechnol Adv 31: 1109-1119
Ditylenchus destructor	ditylenchus_destructor.PRJNA312427.WBPS12	Nematoda	IV	Parasitic: Plant	WormBase
Heterorhabditis bacteriophora	heterorhabditis_bacteriophora.PRJNA13977.WBPS12	Nematoda	V	Free-living	WormBase
Panagrellus redivivus	panagrellus_redivivus.PRJNA186477.WBPS12	Nematoda	IV	Free-living	WormBase
Parastrongyloides trichosuri	parastrongyloides_trichosuri.PRJEB515.WBPS12	Nematoda	IV	Parasitic: Vertebrate	WormBase
Rhabditophanes kr3021	rhabditophanes_kr3021.PRJEB1297.WBPS12	Nematoda	IV	Free-living	WormBase
Strongyloides papillosus	strongyloides_papillosus.PRJEB525.WBPS12	Nematoda	IV	Parasitic: Vertebrate	WormBase
Strongyloides stercoralis	strongyloides_stercoralis.PRJEB528.WBPS12	Nematoda	IV	Parasitic: Vertebrate	WormBase
Strongyloides venezuelensis	strongyloides_venezuelensis.PRJEB530.WBPS12	Nematoda	IV	Parasitic: Vertebrate	WormBase
Xenopus tropicalis	Xenopus_tropicalis.JGI_4.2.pep.all.fa	Amphibian	N/A	Free-living	ENSEMBL Metazoa
Helobdella robusta	Helobdella_robusta.Helro1.pep.all.fa	Annelida	N/A	Free-living	ENSEMBL Metazoa
Gallus gallus	Gallus_gallus.Gallus_gallus-5.0.pep.all.fa	Avian	N/A	Free-living	ENSEMBL Metazoa
Sarcoptes scabiei	Sarcoptes_scabiei.SscaA1.pep.all.fa	Chelicerata	N/A	Parasitic: Vertebrate	ENSEMBL Metazoa
Homo sapiens	Homo_sapiens.GRCh38.pep.all.fa	Chordata	N/A	Free-living	ENSEMBL Metazoa
Mus musculus	Mus_musculus.GRCm38.pep.all.fa	Chordata	N/A	Free-living	ENSEMBL Metazoa
Danio rerio	Danio_rerio.GRCz11.pep.all.fa	Chordata (fish)	N/A	Free-living	ENSEMBL Metazoa
Eptatretus burgeri	Eptatretus_burgeri.Eburgeri_3.2.pep.all.fa	Chordata (hagfish)	N/A	Free-living	ENSEMBL Metazoa
Nematostella vectensis	Nematostella_vectensis.ASM20922v1.pep.all.fa	Cnidaria	N/A	Free-living	ENSEMBL Metazoa
Daphnia pulex	Daphnia_pulex.V1.0.pep.all.fa	Crustacea	N/A	Free-living	ENSEMBL Metazoa
Mnemiopsis leidyi	Mnemiopsis_leidyi.MneLei_Aug2011.pep.all.fa	Ctenophora	N/A	Free-living	ENSEMBL Metazoa
Aedes aegypti	Aedes_aegypti.AaegL3.pep.all.fa	Diptera	N/A	Parasitic: Vertebrate	ENSEMBL Metazoa
Drosophila melanogaster	Drosophila_melanogaster.BDGP6.pep.all.fa	Diptera	N/A	Free-living	ENSEMBL Metazoa
Strongylocentrotus purpuratus	Strongylocentrotus_purpuratus.Spur_3.1.pep.all.fa	Echinodermata	N/A	Free-living	ENSEMBL Metazoa
Apis mellifera	Apis_mellifera.Amel_4.5.pep.all.fa	Hymenoptera	N/A	Free-living	ENSEMBL Metazoa
Bombyx mori	Bombyx_mori.ASM15162v1.pep.all.fa	Lepidoptera	N/A	Free-living	ENSEMBL Metazoa
Crassostrea gigas	Crassostrea_gigas.oyster_v9.pep.all.fa	Mollusca	N/A	Free-living	ENSEMBL Metazoa
Trichoplax adhaerens	Trichoplax_adhaerens.ASM15027v1.pep.all.fa	Placozoa	N/A	Free-living	ENSEMBL Metazoa
Schistosoma mansoni	Schistosoma_mansoni.ASM23792v2.pep.all.fa	Platyhelminthes	N/A	Parasitic: Vertebrate	ENSEMBL Metazoa
Amphimedon queenslandica	Amphimedon_queenslandica.Aqu1.pep.all.fa	Porifera	N/A	Free-living	ENSEMBL Metazoa
Adineta vaga	Adineta_vaga.AMS_PRJEB1171_v1.pep.all.fa	Rotifera	N/A	Free-living	ENSEMBL Metazoa
Ciona intestinalis	Ciona_intestinalis.KH.pep.all.fa	Tunicata	N/A	Free-living	ENSEMBL Metazoa
Saccharomyces cerevisiae	Saccharomyces_cerevisiae.R64-1-1.pep.all.fa	Yeast	N/A	Free-living	ENSEMBL Metazoa

Supplementary Table 2: IDs and accession numbers for all BCL-2 sequences used in the analysis.

Sequence ID	Species	Database	Protein accession number	Phylum	Taxonomic group
BCL_00244	Acanthocheilonema viteae	acanthocheilonema_viteae_prjeb4306	nAv.1.0.1.g05327	Nematoda	Ecdysozoa
BCL_00251	Acanthocheilonema viteae	acanthocheilonema_viteae_prjeb4306	nAv.1.0.1.g05326	Nematoda	Ecdysozoa
BCL_00001	Adineta vaga	Adineta_vaga.AMS_PRJEB1171_v1	GSADVT00001679001	Rotifera	Lophotrochozoa
BCL_00002	Adineta vaga	Adineta_vaga.AMS_PRJEB1171_v1	GSADVT00020536001	Rotifera	Lophotrochozoa
BCL_00006	Aedes aegypti	Aedes_aegypti.AaegL3	AAEL001521-PA	Arthropoda	Ecdysozoa
BCL_00007	Aedes aegypti	Aedes_aegypti.AaegL3	AAEL001515-PA	Arthropoda	Ecdysozoa
BCL_00008	Amphimedon queenslandica	Amphimedon_queenslandica.Aqu1	Aqu2.1.41537_001	Porifera	Porifera
BCL_00009	Amphimedon queenslandica	Amphimedon_queenslandica.Aqu1	Aqu2.1.24557_001	Porifera	Porifera
BCL_00010	Amphimedon queenslandica	Amphimedon_queenslandica.Aqu1	Aqu2.1.28273_001	Porifera	Porifera
BCL_00011	Amphimedon queenslandica	Amphimedon_queenslandica.Aqu1	Aqu2.1.41536_001	Porifera	Porifera
BCL_00364	Amphimedon queenslandica	Amphimedon_queenslandica	I1FWY2_AMPQE	Porifera	Porifera
BCL_00204	Ancylostoma caninum	ancylostoma_caninum_prjna72585	ANCCAN_03779	Nematoda	Ecdysozoa
BCL_00361	Ancylostoma ceylanicum	Bcl_Ancce	EPB77945.1	Nematoda	Ecdysozoa
BCL_00196	Ancylostoma duodenale	ancylostoma_duodenale_prjna72581	ANCDUO_16938	Nematoda	Ecdysozoa
BCL_00215	Angiostrongylus cantonensis	angiostrongylus_cantonensis_prjeb493	ACAC_0000786901	Nematoda	Ecdysozoa
BCL_00195	Anisakis simplex	anisakis_simplex_prjeb496	ASIM_0001721901	Nematoda	Ecdysozoa
BCL_00014	Apis mellifera	Apis_mellifera.Amel_4.5	GB49154-PA	Arthropoda	Ecdysozoa
BCL_00015	Apis mellifera	Apis_mellifera.Amel_4.5	GB41042-PA	Arthropoda	Ecdysozoa
BCL_00376	Arion vulgaris	Arion vulgaris	A0A0B6YFF8_ARIVU	Mollusca	Lophotrochozoa
BCL_00377	Arion vulgaris	Arion vulgaris	A0A0B7BHZ5_ARVUL	Mollusca	Lophotrochozoa
BCL_00206	Ascaris lumbricoides	ascaris_lumbricoides_prjeb4950	ALUE_0001545301	Nematoda	Ecdysozoa
BCL_00220	Ascaris suum	ascaris_suum_prjna62057	AgR017_g161	Nematoda	Ecdysozoa
BCL_00379	Biomphalaria glabrata	Biomphalaria glabrata	A0A182YU15_BIOGL	Mollusca	Lophotrochozoa
BCL_00016	Brugia malayi	Brugia_malayi.Bmal-4.0	Bm5640	Nematoda	Ecdysozoa
BCL_00017	Brugia malayi	Brugia_malayi.Bmal-4.0	Bm14668	Nematoda	Ecdysozoa
BCL_00209a	Brugia pahangi	brugia_pahangi_prjeb497	BPAG_0001210401	Nematoda	Ecdysozoa
BCL_00209b	Brugia pahangi	brugia_pahangi_prjeb497	BPAG_0001210401	Nematoda	Ecdysozoa
BCL_00337	Brugia timori	B_timori_C	A0A0R3QMM0_9BILA	Nematoda	Ecdysozoa
BCL_00336	Brugia timori	B_timori_N	A0A0R3QMM0_9BILA	Nematoda	Ecdysozoa
BCL_00218	Caenorhabditis angaria	caenorhabditis_angaria_prjna51225	Cang_2012_03_13_00245.g7795	Nematoda	Ecdysozoa
BCL_00217	Caenorhabditis brenneri	caenorhabditis_brenneri_prjna20035	WBGene00219366	Nematoda	Ecdysozoa
BCL_00018	Caenorhabditis briggsae	Caenorhabditis_briggsae.CB4	CBG24606	Nematoda	Ecdysozoa
BCL_00343	Caenorhabditis sp.	Caenorhabditis sp.	N/A	Nematoda	Ecdysozoa
BCL_00292	Caenorhabditis elegans	caenorhabditis_elegans_prjna13758	WBGene00000423_ced-9	Nematoda	Ecdysozoa
BCL_00213	Caenorhabditis nigoni	caenorhabditis_nigoni_prjna384657	Cni-ced-9	Nematoda	Ecdysozoa
BCL_00191	Caenorhabditis sinica	caenorhabditis_sinica_prjna194557	Csp5_scaffold_00332.g9574	Nematoda	Ecdysozoa
BCL_00208	Caenorhabditis tropicalis	caenorhabditis_tropicalis_prjna53597	Csp11.Scaffold628.g7399	Nematoda	Ecdysozoa
BCL_00375	Capitella teleta	Capitella teleta	R7TIZ4_CAPTE	Annelida	Lophotrochozoa
BCL_00019	Ciona intestinalis	Ciona_intestinalis.KH	ENSCINP00000024813.2	Chordata	Deuterostomata
BCL_00020	Ciona intestinalis	Ciona_intestinalis.KH	ENSCINP00000000654.3	Chordata	Deuterostomata
BCL_00021	Ciona intestinalis	Ciona_intestinalis.KH	ENSCINP00000019812.3	Chordata	Deuterostomata
BCL_00023	Crassostrea gigas	Crassostrea_gigas.oyster_v9	EKC19322	Mollusca	Lophotrochozoa
BCL_00024	Crassostrea gigas	Crassostrea_gigas.oyster_v9	EKC20695	Mollusca	Lophotrochozoa
BCL_00025	Crassostrea gigas	Crassostrea_gigas.oyster_v9	EKC30554	Mollusca	Lophotrochozoa
BCL_00027	Crassostrea gigas	Crassostrea_gigas.oyster_v9	EKC42310	Mollusca	Lophotrochozoa
BCL_00028	Crassostrea gigas	Crassostrea_gigas.oyster_v9	EKC18663	Mollusca	Lophotrochozoa
BCL_00029	Danio rerio	Danio_rerio.GRCz11	ENSDARP00000027919.6	Chordata	Deuterostomata
BCL_00031	Danio rerio	Danio_rerio.GRCz11	ENSDARP00000124838.2	Chordata	Deuterostomata
BCL_00032	Danio rerio	Danio_rerio.GRCz11	ENSDARP00000120749.2	Chordata	Deuterostomata
BCL_00033	Danio rerio	Danio_rerio.GRCz11	ENSDARP00000023687.7	Chordata	Deuterostomata
BCL_00034	Danio rerio	Danio_rerio.GRCz11	ENSDARP00000108208.2	Chordata	Deuterostomata
BCL_00035	Danio rerio	Danio_rerio.GRCz11	ENSDARP00000139602.1	Chordata	Deuterostomata
BCL_00036	Danio rerio	Danio_rerio.GRCz11	ENSDARP00000141657.2	Chordata	Deuterostomata
BCL_00038	Danio rerio	Danio_rerio.GRCz11	ENSDARP00000098601.3	Chordata	Deuterostomata
BCL_00039	Danio rerio	Danio_rerio.GRCz11	ENSDARP00000124404.1	Chordata	Deuterostomata
BCL_00040	Danio rerio	Danio_rerio.GRCz11	ENSDARP00000123352.1	Chordata	Deuterostomata
BCL_00041	Danio rerio	Danio_rerio.GRCz11	ENSDARP00000151605.1	Chordata	Deuterostomata
BCL_00043	Danio rerio	Danio_rerio.GRCz11	ENSDARP00000040899.6	Chordata	Deuterostomata
BCL_00044	Danio rerio	Danio_rerio.GRCz11	ENSDARP00000126185.1	Chordata	Deuterostomata
BCL_00046	Daphnia pulex	Daphnia_pulex.V1.0	EFX88872	Crustacea	Ecdysozoa
BCL_00047	Daphnia pulex	Daphnia_pulex.V1.0	EFX86309	Crustacea	Ecdysozoa
BCL_00359	Dictyocaulus filaria	Dictyocaulus filaria	Dfil17653233	Nematoda	Ecdysozoa
BCL_00358	Dictyocaulus viviparus	D_vivparus	KJH48938.1|	Nematoda	Ecdysozoa
BCL_00347	Dirofilaria immitis	K31_D_immitis	K31	Nematoda	Ecdysozoa
BCL_00348	Dirofilaria immitis	K43_D_immitis	K43	Nematoda	Ecdysozoa
BCL_00210	Dracunculus medinensis	dracunculus_medinensis_prjeb500	DME_0000043001	Nematoda	Ecdysozoa
BCL_00048	Drosophila melanogaster	Drosophila_melanogaster.BDGP6	FBpp0087182	Arthropoda	Ecdysozoa
BCL_00049	Drosophila melanogaster	Drosophila_melanogaster.BDGP6	FBpp0085443	Arthropoda	Ecdysozoa
BCL_00339	Elaeophora elaphi c	E_elaphi_C	A0A0R3S0K5_9BILA	Nematoda	Ecdysozoa
BCL_00338	Elaeophora elaphi n	E_elaphi_N	A0A0R3S0K5_9BILA	Nematoda	Ecdysozoa
BCL_00253	Enterobius vermicularis	enterobius_vermicularis_prjeb503	EVEC_0000231101	Nematoda	Ecdysozoa
BCL_00051	Eptatretus burgeri	Eptatretus_burgeri.Eburgeri_3.2	ENSEBUP00000003282.1	Chordata	Deuterostomata
BCL_00053	Eptatretus burgeri	Eptatretus_burgeri.Eburgeri_3.2	ENSEBUP00000022387.1	Chordata	Deuterostomata
BCL_00054	Eptatretus burgeri	Eptatretus_burgeri.Eburgeri_3.2	ENSEBUP00000005075.1	Chordata	Deuterostomata
BCL_00057	Eptatretus burgeri	Eptatretus_burgeri.Eburgeri_3.2	ENSEBUP00000014561.1	Chordata	Deuterostomata
BCL_00058	Eptatretus burgeri	Eptatretus_burgeri.Eburgeri_3.2	ENSEBUP00000019353.1	Chordata	Deuterostomata
BCL_00060	Eptatretus burgeri	Eptatretus_burgeri.Eburgeri_3.2	ENSEBUP00000010211.1	Chordata	Deuterostomata
BCL_00061	Eptatretus burgeri	Eptatretus_burgeri.Eburgeri_3.2	ENSEBUP00000022065.1	Chordata	Deuterostomata
BCL_00062	Eptatretus burgeri	Eptatretus_burgeri.Eburgeri_3.2	ENSEBUP00000020968.1	Chordata	Deuterostomata
BCL_00065	Gallus gallus	Gallus_gallus.Gallus_gallus-5.0	ENSGALP00000054103.1	Chordata	Deuterostomata
BCL_00068	Gallus gallus	Gallus_gallus.Gallus_gallus-5.0	ENSGALP00000061728.1	Chordata	Deuterostomata
BCL_00070	Gallus gallus	Gallus_gallus.Gallus_gallus-5.0	ENSGALP00000055311.1	Chordata	Deuterostomata
BCL_00071	Gallus gallus	Gallus_gallus.Gallus_gallus-5.0	ENSGALP00000060382.1	Chordata	Deuterostomata
BCL_00073	Gallus gallus	Gallus_gallus.Gallus_gallus-5.0	ENSGALP00000009257.4	Chordata	Deuterostomata
BCL_00074	Gallus gallus	Gallus_gallus.Gallus_gallus-5.0	ENSGALP00000053183.1	Chordata	Deuterostomata
BCL_00075	Gallus gallus	Gallus_gallus.Gallus_gallus-5.0	ENSGALP00000010509.2	Chordata	Deuterostomata
BCL_00077	Gallus gallus	Gallus_gallus.Gallus_gallus-5.0	ENSGALP00000021251.5	Chordata	Deuterostomata
BCL_00366	Geodia cydonium	Geodia cydonium	Q967D2	Porifera	Porifera
BCL_00357	Haemonchus contortus	H_contortus	CDJ98333.1	Nematoda	Ecdysozoa
BCL_00235	Haemonchus placei	haemonchus_placei_prjeb509	HPLM_0001500101	Nematoda	Ecdysozoa
BCL_00250	Heligmosomoides polygyrus	heligmosomoides_polygyrus_prjeb15396	HPOL_0000258301	Nematoda	Ecdysozoa
BCL_00078	Helobdella robusta	Helobdella_robusta.Helro1	HelroP185432	Annelida	Lophotrochozoa
BCL_00079	Helobdella robusta	Helobdella_robusta.Helro1	HelroP79940	Annelida	Lophotrochozoa
BCL_00081	Helobdella robusta	Helobdella_robusta.Helro1	HelroP183772	Annelida	Lophotrochozoa
BCL_00083	Helobdella robusta	Helobdella_robusta.Helro1	HelroP188428	Annelida	Lophotrochozoa
BCL_00085	Helobdella robusta	Helobdella_robusta.Helro1	HelroP188139	Annelida	Lophotrochozoa
BCL_00086	Helobdella robusta	Helobdella_robusta.Helro1	HelroP194205	Annelida	Lophotrochozoa
BCL_00090	Homo sapiens	Homo_sapiens.GRCh38	ENSP00000406203.1	Chordata	Deuterostomata
BCL_00094	Homo sapiens	Homo_sapiens.GRCh38	ENSP00000329623.3	Chordata	Deuterostomata
BCL_00097	Homo sapiens	Homo_sapiens.GRCh38	ENSP00000451701.1	Chordata	Deuterostomata
BCL_00101	Homo sapiens	Homo_sapiens.GRCh38	ENSP00000353878.6	Chordata	Deuterostomata
BCL_00104	Homo sapiens	Homo_sapiens.GRCh38	ENSP00000375744.4	Chordata	Deuterostomata
BCL_00108	Homo sapiens	Homo_sapiens.GRCh38	ENSP00000477624.1	Chordata	Deuterostomata
BCL_00109	Homo sapiens	Homo_sapiens.GRCh38	ENSP00000335250.6	Chordata	Deuterostomata
BCL_00114	Homo sapiens	Homo_sapiens.GRCh38	ENSP00000314132.3	Chordata	Deuterostomata
BCL_00118	Homo sapiens	Homo_sapiens.GRCh38	ENSP00000453562.1	Chordata	Deuterostomata
BCL_00380	Homo sapiens	Homo sapiens	B2L13_HUMAN	Chordata	Deuterostomata
BCL_00381	Homo sapiens	Homo sapiens	NP_001357197.1	Chordata	Deuterostomata
BCL_00382	Homo sapiens	Homo sapiens	B2L15_HUMAN	Chordata	Deuterostomata
BCL_00368	Hydra vulgaris	Hydra vulgaris	A7LM79_HYDVU	Cnidaria	Cnidaria
BCL_00369	Hydra vulgaris	Hydra vulgaris	A1E3K6_HYDVU	Cnidaria	Cnidaria

BCL_00370	Hydra vulgaris	Hydra vulgaris	A7LM80_HYDVU	Cnidaria	Cnidaria
BCL_00371	Hydra vulgaris	Hydra vulgaris	BAX_HYDVU	Cnidaria	Cnidaria
BCL_00372	Hydra vulgaris	Hydra vulgaris	A7LM81_HYDVU	Cnidaria	Cnidaria
BCL_00373	Hydra vulgaris	Hydra vulgaris	BAK1_HYDVU	Cnidaria	Cnidaria
BCL_00246	Litomosoides sigmodontis	litomosoides_sigmodontis_prjeb3075	nLs.2.1.2.g07653	Nematoda	Ecdysozoa
BCL_00349	Litomosoides sigmodontis	L_sigmonditis2_4	c=33678	Nematoda	Ecdysozoa
BCL_00121	Loa loa	Loa_loa.Loa_loa_V3	EFO25659.1	Nematoda	Ecdysozoa
BCL_00249	Loa loa	loa_loa_prjna246086	EN70_11597	Nematoda	Ecdysozoa
BCL_00378	Lottia gigantea	Lottia gigantea	V3ZZU7_LOTGI	Mollusca	Lophotrochozoa
BCL_00122	Mnemiopsis leidyi	Mnemiopsis_leidyi.MneLei_Aug2011	ML10941a-PA	Ctenophora	Ctenophora
BCL_00126	Mus musculus	Mus_musculus.GRCm38	ENSMUSP00000007803.5	Chordata	Deuterostomata
BCL_00129	Mus musculus	Mus_musculus.GRCm38	ENSMUSP00000108371.1	Chordata	Deuterostomata
BCL_00131	Mus musculus	Mus_musculus.GRCm38	ENSMUSP00000022806.3	Chordata	Deuterostomata
BCL_00133	Mus musculus	Mus_musculus.GRCm38	ENSMUSP00000148265.1	Chordata	Deuterostomata
BCL_00136	Mus musculus	Mus_musculus.GRCm38	ENSMUSP00000135915.1	Chordata	Deuterostomata
BCL_00138	Mus musculus	Mus_musculus.GRCm38	ENSMUSP00000096086.2	Chordata	Deuterostomata
BCL_00139	Mus musculus	Mus_musculus.GRCm38	ENSMUSP00000077757.5	Chordata	Deuterostomata
BCL_00140	Mus musculus	Mus_musculus.GRCm38	ENSMUSP00000096087.2	Chordata	Deuterostomata
BCL_00141	Mus musculus	Mus_musculus.GRCm38	ENSMUSP00000027499.6	Chordata	Deuterostomata
BCL_00143	Mus musculus	Mus_musculus.GRCm38	ENSMUSP00000065612.4	Chordata	Deuterostomata
BCL_00146	Mus musculus	Mus_musculus.GRCm38	ENSMUSP00000034709.5	Chordata	Deuterostomata
BCL_00151	Nematostella vectensis	Nematostella_vectensis.ASM20922v1	EDO39531	Cnidaria	Cnidaria
BCL_00152	Nematostella vectensis	Nematostella_vectensis.ASM20922v1	EDO37464	Cnidaria	Cnidaria
BCL_00153	Nematostella vectensis	Nematostella_vectensis.ASM20922v1	EDO34312	Cnidaria	Cnidaria
BCL_00155	Nematostella vectensis	Nematostella_vectensis.ASM20922v1	EDO42634	Cnidaria	Cnidaria
BCL_00156	Nematostella vectensis	Nematostella_vectensis.ASM20922v1	EDO49892	Cnidaria	Cnidaria
BCL_00161	Nematostella vectensis	Nematostella_vectensis.ASM20922v1	EDO36803	Cnidaria	Cnidaria
BCL_00367	Nematostella vectensis	Nematostella vectensis	A7T5K5_NEMVE	Cnidaria	Cnidaria
BCL_00234	Nippostrongylus brasiliensis	nippostrongylus_brasiliensis_prjeb511	NBR_0001228301	Nematoda	Ecdysozoa
BCL_00360	Oesophagostomum dentatum	O_dentatum	Oesophagostomum	Nematoda	Ecdysozoa
BCL_00284	Onchocerca flexuosa	onchocerca_flexuosa_prjeb512	OFLC_0001586601	Nematoda	Ecdysozoa
BCL_00289	Onchocerca ochengi	onchocerca_ochengi_prjeb1204	OOCN_0000639601	Nematoda	Ecdysozoa
BCL_00163	Onchocerca volvulus	Onchocerca_volvulus.ASM49940v2	OVOC8049	Nematoda	Ecdysozoa
BCL_00272	Oscheius tipulae	oscheius_tipulae_prjeb15512	OTIPU.nOt.2.0.1.g12983	Nematoda	Ecdysozoa
BCL_00299	Parascaris univalens	parascaris_univalens_prjna386823	PgR008_g167	Nematoda	Ecdysozoa
BCL_00268	Plectus sambesii	plectus_sambesii_prjna390260	PSAMB.scaffold906size38830.g9569	Nematoda	Ecdysozoa
BCL_00270	Plectus sambesii	plectus_sambesii_prjna390260	PSAMB.scaffold1669size28877.g14331	Nematoda	Ecdysozoa
BCL_00298	Plectus sambesii	plectus_sambesii_prjna390260	PSAMB.scaffold3237size19146.g20810	Nematoda	Ecdysozoa
BCL_00304	Plectus sambesii	plectus_sambesii_prjna390260	PSAMB.scaffold12479size2746.g34874	Nematoda	Ecdysozoa
BCL_00311	Plectus sambesii	plectus_sambesii_prjna390260	PSAMB.scaffold391size53556.g5437	Nematoda	Ecdysozoa
BCL_00276	Pristionchus exspectatus	pristionchus_exspectatus_prjeb6009	scaffold118-EXSNAP2012.46	Nematoda	Ecdysozoa
BCL_00291	Pristionchus pacificus	pristionchus_pacificus_prjna12644	WBGene0010942	Nematoda	Ecdysozoa
BCL_00221	Romanomermis culicivorax	romanomermis_culicivorax_prjeb1358	nRc.2.0.1.g00477	Nematoda	Ecdysozoa
BCL_00236	Romanomermis culicivorax	romanomermis_culicivorax_prjeb1358	nRc.2.0.1.g46209	Nematoda	Ecdysozoa
BCL_00243	Romanomermis culicivorax	romanomermis_culicivorax_prjeb1358	nRc.2.0.1.g07841	Nematoda	Ecdysozoa
BCL_00256	Romanomermis culicivorax	romanomermis_culicivorax_prjeb1358	nRc.2.0.1.g42278	Nematoda	Ecdysozoa
BCL_00165	Sarcoptes scabiei	Sarcoptes_scabiei.SscaA1	SSCA004373-PA	Arthropoda	Ecdysozoa
BCL_00166	Sarcoptes scabiei	Sarcoptes_scabiei.SscaA1	SSCA002936-PA	Arthropoda	Ecdysozoa
BCL_00168	Schistosoma mansoni	Schistosoma_mansoni.ASM23792v2	Smp_095190.1:pep	Platyhelminthes	Lophotrochozoa
BCL_00169	Schistosoma mansoni	Schistosoma_mansoni.ASM23792v2	Smp_072180.1:pep	Platyhelminthes	Lophotrochozoa
BCL_00297	Soboliphyme baturini	soboliphyme_baturini_prjeb516	SBAD_0001034701	Nematoda	Ecdysozoa
BCL_00223	Steinernema carpocapsae	steinernema_carpocapsae_prjna202318	L596_g20916	Nematoda	Ecdysozoa
BCL_00229	Steinernema carpocapsae	steinernema_carpocapsae_prjna202318	L596_g23187	Nematoda	Ecdysozoa
BCL_00222	Steinernema scapterisci	steinernema_scapterisci_prjna204942	L892_g11635	Nematoda	Ecdysozoa
BCL_00170	Strongylocentrotus purpuratus	Strongylocentrotus_purpuratus.Spur_3.1	SPU_024469-tr	Echinodermata	Deuterostomata
BCL_00171	Strongylocentrotus purpuratus	Strongylocentrotus_purpuratus.Spur_3.1	SPU_010786-tr	Echinodermata	Deuterostomata
BCL_00172	Strongylocentrotus purpuratus	Strongylocentrotus_purpuratus.Spur_3.1	SPU_014028-tr	Echinodermata	Deuterostomata
BCL_00173	Strongylocentrotus purpuratus	Strongylocentrotus_purpuratus.Spur_3.1	SPU_006124-tr	Echinodermata	Deuterostomata
BCL_00174	Strongylocentrotus purpuratus	Strongylocentrotus_purpuratus.Spur_3.1	SPU_010641-tr	Echinodermata	Deuterostomata
BCL_00175	Strongylocentrotus purpuratus	Strongylocentrotus_purpuratus.Spur_3.1	SPU_021416-tr	Echinodermata	Deuterostomata
BCL_00177	Strongylocentrotus purpuratus	Strongylocentrotus_purpuratus.Spur_3.1	SPU_028607-tr	Echinodermata	Deuterostomata
BCL_00178	Strongylocentrotus purpuratus	Strongylocentrotus_purpuratus.Spur_3.1	SPU_001916-tr	Echinodermata	Deuterostomata
BCL_00383	Strongyloides papillosus_PRJEB525	strongyloides_papillosus_PRJEB525	SPAL_0001699500.1	Nematoda	Ecdysozoa
BCL_00384	Strongyloides ratti	Strongyloides_ratti_PRJEB125	SRAE_2000144000	Nematoda	Ecdysozoa
BCL_00385	Strongyloides stercoralis	Strongyloides_stercoralis_PRJEB528	SSTP_0000155500.2	Nematoda	Ecdysozoa
BCL_00386	Strongyloides venezuelensis	Strongyloides_venezuelensis_PRJEB530	SVE_0785600.1	Nematoda	Ecdysozoa
BCL_00365	Suberites domuncula	Suberites domuncula	Q9N754_SUBDO	Porifera	Porifera
BCL_00274	Syphacia muris	syphacia_muris_prjeb524	SMUV_0000606901	Nematoda	Ecdysozoa
BCL_00266	Teladorsagia circumcincta	teladorsagia_circumcincta_prjna72569	TELCIR_07472	Nematoda	Ecdysozoa
BCL_00280	Teladorsagia circumcincta	teladorsagia_circumcincta_prjna72569	TELCIR_07409	Nematoda	Ecdysozoa
BCL_00286	Thelazia callipaeda	thelazia_callipaeda_prjeb1205	TCLT_0000694101	Nematoda	Ecdysozoa
BCL_00308	Toxocara canis	toxocara_canis_prjna248777	Tcan_09290	Nematoda	Ecdysozoa
BCL_00307	Trichinella britovi	trichinella_britovi_prjna257433	T03_15422	Nematoda	Ecdysozoa
BCL_00278	Trichinella murrelli	trichinella_murrelli_prjna257433	T05_5053	Nematoda	Ecdysozoa
BCL_00275	Trichinella nativa	trichinella_nativa_prjna257433	T02_5514	Nematoda	Ecdysozoa
BCL_00295	Trichinella nelsoni	trichinella_nelsoni_prjna257433	T07_11990	Nematoda	Ecdysozoa
BCL_00282	Trichinella papuae	trichinella_papuae_prjna257433	T10_12694	Nematoda	Ecdysozoa
BCL_00287	Trichinella patagoniensis	trichinella_patagoniensis_prjna257433	T12_9003	Nematoda	Ecdysozoa
BCL_00324	Trichinella pseudospiralis1	T_pseudospiralis1	A0A0V1G333_TRIPS	Nematoda	Ecdysozoa
BCL_00290	Trichinella spiralis	trichinella_spiralis_prjna257433	T01_5514	Nematoda	Ecdysozoa
BCL_00277	Trichinella t6	trichinella_t6_prjna257433	T06_4072	Nematoda	Ecdysozoa
BCL_00300	Trichinella t8	trichinella_t8_prjna257433	T08_9629	Nematoda	Ecdysozoa
BCL_00296	Trichinella t9	trichinella_t9_prjna257433	T09_15613	Nematoda	Ecdysozoa
BCL_00312	Trichinella zimbabwensis	trichinella_zimbabwensis_prjna257433	T11_787_Bcl2l1	Nematoda	Ecdysozoa
BCL_00182	Trichoplax adhaerens	Trichoplax_adhaerens.ASM15027v1	TriadP7511	Placozoa	Placozoa
BCL_00183	Trichoplax adhaerens	Trichoplax_adhaerens.ASM15027v1	TriadP63759	Placozoa	Placozoa
BCL_00363	Trichostrongylus colubriformis	T_colubriformis	Trichostrongylus	Nematoda	Ecdysozoa
BCL_00269	Trichuris muris	trichuris_muris_prjeb126	WBGene00285384	Nematoda	Ecdysozoa
BCL_00281	Trichuris muris	trichuris_muris_prjeb126	WBGene00295603	Nematoda	Ecdysozoa
BCL_00199	Trichuris suis	trichuris_suis_prjna179528	D918_01910	Nematoda	Ecdysozoa
BCL_00219	Trichuris suis	trichuris_suis_prjna179528	D918_00166	Nematoda	Ecdysozoa
BCL_00288	Trichuris trichiura	trichuris_trichiura_prjeb535	TTRE_0000317601	Nematoda	Ecdysozoa
BCL_00374	Urechis unicinctus	Urechis unicinctus	A0A144KBE2_UREUN	Annelida	Lophotrochozoa
BCL_00271	Wuchereria bancrofti	wuchereria_bancrofti_prjna275548	snap_masked-PairedContig_1052-processed-gene-3.10	Nematoda	Ecdysozoa
BCL_00330	Wuchereria bancrofti	W_bancrofti1	Wucheria	Nematoda	Ecdysozoa
BCL_00184	Xenopus tropicalis	Xenopus_tropicalis.JGI_4.2	ENSXETP00000052056.2	Chordata	Deuterostomata
BCL_00185	Xenopus tropicalis	Xenopus_tropicalis.JGI_4.2	ENSXETP00000053903.1	Chordata	Deuterostomata
BCL_00186	Xenopus tropicalis	Xenopus_tropicalis.JGI_4.2	ENSXETP00000031936.3	Chordata	Deuterostomata
BCL_00187	Xenopus tropicalis	Xenopus_tropicalis.JGI_4.2	ENSXETP00000050759.3	Chordata	Deuterostomata
BCL_00188	Xenopus tropicalis	Xenopus_tropicalis.JGI_4.2	ENSXETP00000063039.1	Chordata	Deuterostomata
BCL_00190	Xenopus tropicalis	Xenopus_tropicalis.JGI_4.2	ENSXETP00000056124.2	Chordata	Deuterostomata
BCL_00328	Xiphinema index	Xiphinema_index		Nematoda	Ecdysozoa
PDB# 5whi_chain A	Homo sapiens (BCL-2A1)			Chordata	Deuterostomata
PDB# 5wdd_chain A	Gallus gallus (BOK)			Chordata	Deuterostomata
PDB# 2yv6_chain A	Homo sapiens (BAK)			Chordata	Deuterostomata
PDB# 1wsx_chain A	Mus musculus (MCL-1)			Chordata	Deuterostomata
PDB# 1pq0_chain A	Mus musculus (BCL-XL)			Chordata	Deuterostomata
PDB# 1g5m_chain A	Homo sapiens (BCL-2)			Chordata	Deuterostomata
PDB# 1f16_chain A	Homo sapiens (BAX)			Chordata	Deuterostomata
PDB# 1ohu_chainA	Caenorhabditis elegans (CED-9)			Nematoda	Ecdysozoa
PDB# 6v4m_chain A	Trichuris suis (BCL-2)			Nematoda	Ecdysozoa

Supplementary Table 3: Pairwise % sequence identitites for BCL-2 proteins in Clades, I, C and III with more than one BCL-2 family member

Clade I/C

Species		Trichuris suis_1	Trichuris suis_2	Trichuris muris_1	Trichuris muris_2	Romanomermis culicivorax_1	Romanomermis culicivorax_2	Romanomermis culicivorax_3	Romanomermis culicivorax_4
	**Sequence ID**	BCL_00219	BCL_00199	BCL_00269	BCL_00281	BCL_00243	BCL_00221	BCL_00236	BCL_00256
Trichuris suis_1	BCL_00219	**100**	27.4	72.6	22.4	10	29.1	16.2	40.7
Trichuris suis_2	BCL_00199	27.4	**100**	26.1	50.5	15	24.7	13.6	29.4
Trichuris muris_1	BCL_00269	72.6	26.1	**100**	19.8	14.8	30.6	15	37.5
Trichuris muris_2	BCL_00281	22.4	50.5	19.8	**100**	16	23.5	16.8	24.5
Romanomermis culicivorax_1	BCL_00243	10	15	14.8	16.9	**100**	13.4	10.1	11.7
Romanomermis culicivorax_2	BCL_00221	29.1	24.7	30.6	23.5	13.4	**100**	15.1	42.2
Romanomermis culicivorax_3	BCL_00236	16.2	13.6	15	16.8	10.1	15.1	**100**	17.8
Romanomermis culicivorax_4	BCL_00256	40.7	29.4	37.5	24.5	11.7	42.2	17.8	**100**
Plectus sambesii_1	BCL_00268	29	22.4	29.1	23.3	15.7	28.2	17.3	32.2
Plectus sambesii_2	BCL_00270	15.8	13.8	14.6	13.4	10.8	14.2	16.9	17
Plectus sambesii_3	BCL_00298	24.5	21.7	24.4	19.7	10.1	27	17.1	28.5
Plectus sambesii_4	BCL_00304	25.2	21.5	26.2	20.8	9.9	27.1	16.7	29.5
Plectus sambesii_5	BCL_00311	19.4	17.4	22.3	15.1	16	18	13.7	21.4

Clade III

Species		Loa loa_1	Loa loa_2	Wuchereria bancrofti_1	Wuchereria bancrofti_2	Elaeophora elaphi_1	Elaeophora elaphi_2	Brugia malayi_1	Brugia malayi_2
	**Sequence ID**	BCL_00249	BCL_00121	BCL_00271	BCL_00330	BCL_00339	BCL_00338	BCL_00016	BCL_00017
Loa loa_1	BCL_00249	**100**	46.4	82	41.6	72.6	35.7	82.4	44.2
Loa loa_2	BCL_00121	46.4	**100**	46.6	71.4	42.1	61.5	47.5	76.8
Wuchereria bancrofti_1	BCL_00271	82	46.6	**100**	41.6	69.4	36.1	96.1	45.4
Wuchereria bancrofti_2	BCL_00330	41.6	71.4	41.6	**100**	38.8	58.1	44.3	85.5
Elaeophora elaphi_1	BCL_00339	72.6	42.1	69.4	38.8	**100**	35.3	69.2	41.7
Elaeophora elaphi_2	BCL_00338	35.7	61.5	36.1	58.1	35.3	**100**	37.7	58.5
Brugia malayi_1	BCL_00016	82.4	47.5	96.1	44.3	69.2	37.7	**100**	46.2
Brugia malayi_2	BCL_00017	44.2	76.8	45.4	85.5	41.7	58.5	46.2	**100**
Brugia pahangi_1	BCL_00209_B	80.3	46.6	94.8	43.4	67.6	36.4	97.4	45.4
Brugia pahangi_2	BCL_00209_A	41.6	71.8	42.5	91.5	39.3	58.1	43.3	91.7
Brugia timori_1	BCL_00337	74.4	43	86.8	40	62.5	34.6	90.3	41.9
Brugia timori_2	BCL_00336	42	72.9	43.3	81.2	39.8	61.1	44	95
Acanthocheilonema viteae_1	BCL_00244	76.5	45.1	73.7	39.3	75	34.7	73.1	42.9
Acanthocheilonema viteae_2	BCL_00251	44.7	75.4	43.6	68.4	39.5	63.8	42.5	72.4
Litomosoides sigmodontis_1	BCL_00349	69.5	43.6	70.5	38.7	70.3	33.7	68.2	43.3
Litomosoides sigmodontis_2	BCL_00246	43	72.4	42.5	64.9	39.5	56.2	43.6	70.6
Dirofilaria immitis_1	BCL_00347	78.5	46.4	77.3	42.2	68.4	36.3	76.8	45.9
Dirofilaria immitis_2	BCL_00348	46.4	73.7	46.2	65.4	42	57.4	47.2	70.6

Plectus sambesii_1	Plectus sambesii_2	Plectus sambesii_3	Plectus sambesii_4	Plectus sambesii_5
BCL_00268	BCL_00270	BCL_00298	BCL_00304	BCL_00311
29	15.8	24.5	25.2	19.4
22.4	13.8	21.7	21.5	17.4
29.1	14.6	24.4	26.2	22.3
23.3	13.4	19.7	20.8	15.1
15.7	10.8	10.1	9.9	16
28.2	14.2	27	27.1	18
17.3	16.9	17.1	16.7	13.7
32.2	17	28.5	29.5	21.4
**100**	19.8	42.4	46.9	23.6
19.8	**100**	15.6	16.4	21.1
42.4	15.6	**100**	73.2	13.8
46.9	16.4	73.2	**100**	14.8
23.6	21.1	13.8	14.8	**100**

Brugia pahangi_1	Brugia pahangi_2	Brugia timori_1	Brugia timori_2	Acanthocheilonema viteae_1	Acanthocheilonema viteae_2	Litomosoides sigmodontis_1	Litomosoides sigmodontis_2	Dirofilaria immitis_1	Dirofilaria immitis_2
BCL_00209_B	BCL_00209_A	BCL_00337	BCL_00336	BCL_00244	BCL_00251	BCL_00349	BCL_00246	BCL_00347	BCL_00348
80.3	41.6	74.4	42	76.5	44.7	69.5	43	78.5	46.4
46.6	71.8	43	72.9	45.1	75.4	43.6	72.4	46.4	73.7
94.8	42.5	86.8	43.3	73.7	43.6	70.5	42.5	77.3	46.2
43.4	91.5	40	81.2	39.3	68.4	38.7	64.9	42.2	65.4
67.6	39.3	62.5	39.8	75	39.5	70.3	39.5	68.4	42
36.4	58.1	34.6	61.1	34.7	63.8	33.7	56.2	36.3	57.4
97.4	43.3	90.3	44	73.1	42.5	68.2	43.6	76.8	47.2
45.4	91.7	41.9	95	42.9	72.4	43.3	70.6	45.9	70.6
**100**	42.5	87.9	43.3	71	42.5	67.4	40.7	74.7	46.4
42.5	**100**	39.2	87.1	40	67.5	40.8	66.2	43.3	64.9
87.9	39.2	**100**	40.1	66	38.5	61.7	39.5	69.4	42.7
43.3	87.1	40.1	**100**	40.9	68.8	41.2	67.1	43.7	67.1
71	40	66	40.9	**100**	41.4	77.4	41.5	75.2	43.8
42.5	67.5	38.5	68.8	41.4	**100**	39.5	74.6	42.7	71.1
67.4	40.8	61.7	41.2	77.4	39.5	**100**	40.5	67.4	40.1
40.7	66.2	39.5	67.1	41.5	74.6	40.5	**100**	42.7	66.7
74.7	43.3	69.4	43.7	75.2	42.7	67.4	42.7	**100**	44.3
46.4	64.9	42.7	67.1	43.8	71.1	40.1	66.7	44.3	**100**

Supplementary Table 4: Key features of Bcl-2 homology (BH) domains. The consensus sequence refers to that found in mammalian BCL-2 family members.

BH domain	Consensus sequence	General features	Nematode features	
BH1	NWGR(ϕ)$_6$F<br>(where ϕ are generally hydrophobic residues)	• Structural role that forms the base of the BH3-ligand binding groove.	N	• Commonly “S” or “T” in all clades though can be an “R” in some Clade I species.
			W	• Aromatic nature is conserved due to role in structurally important hydrophobic interactions with residues in the BH2 domain.<br>• Only conserved in Clade I nematodes<br>• “Y” in all other Clades where the hydroxyl group of the “Y” hydrogen bonds with an aspartate residue only conserved (or substituted with similarly polar residues) in Clade IV and V nematodes.
			GR	• Conserved across all nematode clades<br>• “G” plays an important structural role at the α4- α5 hairpin apex.<br>• “R” makes an important electrostatic interaction with a conserved aspartate on BH3 ligands.
			(ϕ)$_6$F	• Conserved across all nematode clades<br>• “F” sits at the bottom of the BH3 ligand binding groove
BH2	W$_1$I(X)$_3$GGW$_2$(X)$_2$F<br>(where X represents any residue)	• Key hydrophobic residues pack against the BH1 domain and the hydrophobic core of the molecule.	W$_1$, W$_2$, F	• Conserved in all nematode Clades
			I	• Generally, “P”, “K”, or “V” in Clade III, IV and V nematodes. Clade I nematodes conform closely to the mammalian BH2 sequence
			GG	• Commonly “RS” in Clade III, IV and V nematodes. Clade I nematodes conform closely to the mammalian BH2 sequence.<br>• Both “GG” and “RS” motifs connect α7 with α8.
BH3	ϕ_1s$_1$(X)$_2\phi_2$RXϕ_3s$_2$D$_1$(D/E)ϕ_4	• Important structural and functional element of multi-BH3 domain BCL-2 family members.	ϕ_1	• Conserved in Clade I. Generally polar in Clade III, IV and V.
			s1	• Conserved in Clade I. Generally “H”, “Y”, “F” in others

	(where "ϕ" is a hydrophobic residue and "s" is a small amino acid)	• α2 helix on which it is located forms one "wall" of the ligand binding groove.<br>• In BAX and BAK, the BH3 domain enables the formation of homodimers which is critical to their oligomerization and mitochondrial pore formation. The BH3 domain also mediates binding to pro-survival members of the family which inhibits their homodimerization.	ϕ_2	• Frequently "L" in mammalian proteins, generally "M" or "I" in all nematodes.
			R	• Conserved across all nematode clades except Clade IV where it is "Y" or "V"
			ϕ_3	• Large hydrophobic "M" in Clade I nematodes, in contrast to a smaller hydrophobic residue ("L" or "V") in other clades.
			s_2	• Conserved in all clades except Clade IV where it is "T" or "C"
			D_1	• In Clade I nematodes, this is generally "D" or "E".<br>• Replaced with "L" in Clade III nematodes.<br>• Generally polar residues in Clades IV and V nematodes.
			D/E	• Clade I nematodes conform to the negatively charged consensus residue.<br>• Hydrophobic "I" is found in this position in Clades III, IV, and V nematodes.
			ϕ_4	• Conserved as "F" across all nematodes.
BH4	ϕ_1 $\phi_2 X_1 X_2$ ϕ_3 ϕ_4<br>(where "ϕ" is a hydrophobic residue)	• Found on the α1 helix where all the hydrophobic residues and some intervening residues pack against or interact with elements of the BH1, BH2 and BH3 domains.<br>• Readily identifiable through sequence alignments based on available crystal structures of CED-9 {Kvansakul, 2008 #58}	ϕ_1, ϕ_4	• Where the residue is normally a "L" or "I", it is an aromatic ("F" or "Y") in Clade III, IV and V nematodes. Clade I nematodes conform to the typical sequence.
			X_2	• Usually a "D" in nematodes which is a common feature of BH4 domains. This residue makes a salt bridge with a complementarily charged residue on the BH3 domain.

Supplementary Table 5: Crystallographic statistics. Highest-resolution shell data are shown in parentheses.

	***T. suis* BCL-2**<br><br>**PDB: 6V4M**
**DATA COLLECTION**	
Wavelength	0.954
Resolution range	39.2 - 1.599<br>(1.656 - 1.599)
Space group	P 41 21 2
Unit cell	55.248 55.248<br>111.249 90 90 90
Total reflections	332522 (52936)
Unique reflections	23576 (3723)
Multiplicity	14.1 (14.2)
Completeness (%)	99.99 (100.00)
Mean I/sigma(I)	25.14 (3.94)
R-merge	0.078 (0.77)
CC1/2	0.999 (0.914)
**REFINEMENT**	
Reflections used	23563 (2303)
R-work	0.1688 (0.2165)
R-free	0.1917 (0.2453)
Number of atoms	1449
- macromolecules	1320
- solvent	121
Protein residues	163
RMS (bonds)	0.006
RMS (angles)	0.78
Average B-factor	21.10
- macromolecules	19.93
- solvent	30.51

Supplementary Table 6: List of aligned proteins used to construct the APAF-1 phylogenetic tree

Gene label	Protein database (See Supplementary Table 1)	Protein ID
APF_20001	acanthocheilonema_viteae.PRJEB4306.WBPS12	nAv.1.0.1.t03675-RA
APF_20005	ancylostoma_ceylanicum.PRJNA72583.WBPS12	maker-ANCCEYDFT_Contig1667-pred_gff_snap-gene-0.4-mRNA-1
APF_20008	angiostrongylus_costaricensis.PRJEB494.WBPS12	ACOC_0000131601-mRNA-1
APF_20010	ascaris_lumbricoides.PRJEB4950.WBPS12	ALUE_0000726401-mRNA-1
APF_20011	ascaris_suum.PRJNA62057.WBPS12	AgR005_g368_t03
APF_20013	brugia_malayi.PRJNA10729.WBPS12	Bm2618
APF_20015	brugia_timori.PRJEB4663.WBPS12	BTMF_0001328501-mRNA-1
APF_20016	caenorhabditis_angaria.PRJNA51225.WBPS12	Cang_2012_03_13_00081.g3732.t3
APF_20017	caenorhabditis_brenneri.PRJNA20035.WBPS12	CBN29155
APF_20018	caenorhabditis_briggsae.PRJNA10731.WBPS12	CBG17963b
APF_20019	caenorhabditis_elegans.PRJNA13758.WBPS12	C35D10.9a
APF_20020	caenorhabditis_japonica.PRJNA12591.WBPS12	CJA24916
APF_20021	caenorhabditis_latens.PRJNA248912.WBPS12	FL83_21473
APF_20022	caenorhabditis_nigoni.PRJNA384657.WBPS12	Cni-ced-4
APF_20024	caenorhabditis_remanei.PRJNA248911.WBPS12	FL82_10034
APF_20026	caenorhabditis_sinica.PRJNA194557.WBPS12	Csp5_scaffold_00455.g11603.t1
APF_20027	caenorhabditis_tropicalis.PRJNA53597.WBPS12	Csp11.Scaffold593.g5186.t1
APF_20029	dictyocaulus_viviparus.PRJEB5116.WBPS12	nDv.1.0.1.t04992
APF_20031	diploscapter_pachys.PRJNA280107.WBPS12	WR25_20290.1
APF_20032	dirofilaria_immitis.PRJEB1797.WBPS12	nDi.2.2.2.t09169
APF_20033	ditylenchus_destructor.PRJNA312427.WBPS12	Dd_05713
APF_20035	elaeophora_elaphi.PRJEB502.WBPS12	EEL_0001025201-mRNA-1
APF_20036	enterobius_vermicularis.PRJEB503.WBPS12	EVEC_0000222001-mRNA-1
APF_20038	globodera_rostochiensis.PRJEB13504.WBPS12	GROS_g12548.t1
APF_20040	haemonchus_contortus.PRJEB506.WBPS12	HCON_00074560
APF_20044	heligmosomoides_polygyrus.PRJEB15396.WBPS12	HPOL_0001549201-mRNA-1
APF_20045	heterorhabditis_bacteriophora.PRJNA13977.WBPS12	Hba_19802
APF_20046	litomosoides_sigmodontis.PRJEB3075.WBPS12	nLs.2.1.2.t03969-RA
APF_20047	loa_loa.PRJNA246086.WBPS12	EN70_11943
APF_20049	meloidogyne_arenaria.PRJEB8714.WBPS12	M.Arenaria_Scaff161g004386
APF_20051	meloidogyne_floridensis.PRJEB6016.WBPS12	augustus_masked-nMf.1.1.scaf00378-processed-gene-0.1-mRNA-1
APF_20052	meloidogyne_graminicola.PRJNA411966.WBPS12	NXFT01004011.1.10461_g
APF_20055	meloidogyne_javanica.PRJEB8714.WBPS12	M.Javanica_Scaff4487g034856
APF_20058	oesophagostomum_dentatum.PRJNA72579.WBPS12	OESDEN_14926
APF_20060	onchocerca_flexuosa.PRJNA230512.WBPS12	X798_04507
APF_20061	onchocerca_ochengi.PRJEB1204.WBPS12	OOCN_0000695801-mRNA-1
APF_20063	onchocerca_volvulus.PRJEB513.WBPS12	OVOC7562
APF_20064	oscheius_tipulae.PRJEB15512.WBPS12	OTIPU.nOt.2.0.1.t12697
APF_20065	panagrellus_redivivus.PRJNA186477.WBPS12	Pan_g7422.t1
APF_20067	parascaris_univalens.PRJNA386823.WBPS12	PgR007_g174_t02
APF_20068	parastrongyloides_trichosuri.PRJEB515.WBPS12	PTRK_0001410100.1
APF_20070	pristionchus_exspectatus.PRJEB6009.WBPS12	scaffold16-EXSNAP2012.32
APF_20072	rhabditophanes_kr3021.PRJEB1297.WBPS12	RSKR_0000940950.1
APF_20075	steinernema_carpocapsae.PRJNA202318.WBPS12	L596_g26645.t1
APF_20076	steinernema_feltiae.PRJNA204661.WBPS12	L889_g24289.t1
APF_20077	steinernema_glaseri.PRJNA204943.WBPS12	L893_g33705.t1
APF_20079	steinernema_scapterisci.PRJNA204942.WBPS12	L892_g25405.t1
APF_20080	strongyloides_papillosus.PRJEB525.WBPS12	SPAL_0000267200.1
APF_20081	strongyloides_ratti.PRJEB125.WBPS12	SRAE_1000266300
APF_20082	strongyloides_stercoralis.PRJEB528.WBPS12	SSTP_0000599200.1
APF_20083	strongyloides_venezuelensis.PRJEB530.WBPS12	SVE_1899400.1
APF_20085	syphacia_muris.PRJEB524.WBPS12	SMUV_0000494901-mRNA-1
APF_20087	thelazia_callipaeda.PRJEB1205.WBPS12	TCLT_0000234601-mRNA-1
APF_20089	toxocara_canis.PRJNA248777.WBPS12	Tcan_08044.1
APF_20090	trichinella_britovi.PRJNA257433.WBPS12	T03_10714.6
APF_20091	trichinella_murrelli.PRJNA257433.WBPS12	T05_1252.1
APF_20092	trichinella_nativa.PRJNA179527.WBPS12	D917_01214
APF_20094	trichinella_nelsoni.PRJNA257433.WBPS12	T07_13442.3
APF_20095	trichinella_papuae.PRJNA257433.WBPS12	T10_5087.1
APF_20096	trichinella_patagoniensis.PRJNA257433.WBPS12	T12_8366.1
APF_20097	trichinella_spiralis.PRJNA12603.WBPS12	EFV57474
APF_20099	trichinella_t6.PRJNA257433.WBPS12	T06_5140.1
APF_20101	trichinella_t9.PRJNA257433.WBPS12	T09_540.5
APF_20102	trichinella_zimbabwensis.PRJNA257433.WBPS12	T11_5223.4
APF_20103	trichuris_muris.PRJEB126.WBPS12	TMUE_2000007907
APF_20105	trichuris_suis.PRJNA208415.WBPS12	M51309155
APF_20108	wuchereria_bancrofti.PRJEB536.WBPS12	WBA_0000526201-mRNA-1
APF_20111	Aedes_aegypti.AaegL3	AAEL000874-PA
APF_20112	Amphimedon_queenslandica.Aqu1	Aqu2.1.23762_001
APF_20113	Apis_mellifera.Amel_4.5	GB52453-PA
APF_20114	Bombyx_mori.ASM15162v1	BGIBMGA011028-TA
APF_20123	Danio_rerio.GRCz11	ENSDARP00000008347.6
APF_20124	Daphnia_pulex.V1.0	EFX74325
APF_20125	Drosophila_melanogaster.BDGP6	FBpp0086122
APF_20126	Eptatretus_burgeri.Eburgeri_3.2	ENSEBUP00000005872.1
APF_20127	Gallus_gallus.Gallus_gallus-5.0	ENSGALP00000037437.2
APF_20129	Homo_sapiens.GRCh38	ENSP00000353059.2
APF_20132	Mus_musculus.GRCm38	ENSMUSP00000124134.1
APF_20133	Nematostella_vectensis.ASM20922v1	EDO47633
APF_20135	Pristionchus_pacificus.P_pacificus-5.0	PPA02552
APF_20136	Sarcoptes_scabiei.SscaA1	SSCA007724-PA
APF_20137	Schistosoma_mansoni.ASM23792v2	Smp_140260.1:pep
APF_20138	Strongylocentrotus_purpuratus.Spur_3.1	SPU_025776-tr
APF_20141	Trichoplax_adhaerens.ASM15027v1	TriadP61488
APF_20142	Trichoplax_adhaerens.ASM15027v1	TriadP32738
APF_20143	Xenopus_tropicalis.JGI_4.2	ENSXETP00000014654.2
		GSADVT00006516001
