# Supplementary material for: Diversity in the intrinsic apoptosis pathway of nematodes
Source: Commun Biol. 2020 Aug 28;3:478. doi: 10.1038/s42003-020-01208-5 (PMC7456325; doi:10.1038/s42003-020-01208-5)
Supplement: Supplementary file 2 — Description of Additional Supplementary Files [file 42003_2020_1208_MOESM2_ESM.pdf]

Description of Additional Supplementary Files

Supplementary Data 1: Excel spreadsheet for data in Supplementary Table 1

Supplementary Data 2: Excel spreadsheet for data in Supplementary Table 2

Supplementary Data 3: Excel spreadsheet for data in Supplementary Table 6

Supplementary Data 4: Raw data file for Figure 6a
