# Supplementary material for: Diversity in the intrinsic apoptosis pathway of nematodes
Source: Commun Biol. 2020 Aug 28;3:478. doi: 10.1038/s42003-020-01208-5 (PMC7456325; doi:10.1038/s42003-020-01208-5)
Supplement: Supplementary file 8 — Reporting Summary [file 42003_2020_1208_MOESM8_ESM.pdf]

nature research

Corresponding author(s): W. Douglas Fairlie
Erinna F. Lee

Last updated by author(s): 2020/05/06

Reporting Summary

Nature Research wishes to improve the reproducibility of the work that we publish. This form provides structure for consistency and transparency in reporting. For further information on Nature Research policies, see our Editorial Policies and the Editorial Policy Checklist.

Statistics

For all statistical analyses, confirm that the following items are present in the figure legend, table legend, main text, or Methods section.

n/a	Confirmed	
☐	☒	The exact sample size (*n*) for each experimental group/condition, given as a discrete number and unit of measurement
☒	☐	A statement on whether measurements were taken from distinct samples or whether the same sample was measured repeatedly
☒	☐	The statistical test(s) used AND whether they are one- or two-sided<br>*Only common tests should be described solely by name; describe more complex techniques in the Methods section.*
☒	☐	A description of all covariates tested
☒	☐	A description of any assumptions or corrections, such as tests of normality and adjustment for multiple comparisons
☐	☒	A full description of the statistical parameters including central tendency (e.g. means) or other basic estimates (e.g. regression coefficient) AND variation (e.g. standard deviation) or associated estimates of uncertainty (e.g. confidence intervals)
☒	☐	For null hypothesis testing, the test statistic (e.g. *F*, *t*, *r*) with confidence intervals, effect sizes, degrees of freedom and *P* value noted<br>*Give P values as exact values whenever suitable.*
☐	☒	For Bayesian analysis, information on the choice of priors and Markov chain Monte Carlo settings
☒	☐	For hierarchical and complex designs, identification of the appropriate level for tests and full reporting of outcomes
☒	☐	Estimates of effect sizes (e.g. Cohen's *d*, Pearson's *r*), indicating how they were calculated

Our web collection on statistics for biologists contains articles on many of the points above.

Software and code

Policy information about availability of computer code

Data collection	HMMER v.3.2.1; http://hmmer.janelia.org/);
Data analysis	CD-HIT (v.4.7); PROMALS3D ; MUSCLE v.3.7 (-refine option); trimAl v.1.4.1 (-gappyout option); MrBayes (v.3.2.6); ggtree (v.1.10.5), R (v.3.4.3; http://www.R-project.org/); FLOWJO v10.5.3; PRISM v8.2.0; PHENIX v1.11.1; COOT v0.8.9.2; NT.Analysis software v1.5.41; Auto-Rickshaw (http://www.embl-hamburg.de/Auto-Rickshaw/)

For manuscripts utilizing custom algorithms or software that are central to the research but not yet described in published literature, software must be made available to editors and reviewers. We strongly encourage code deposition in a community repository (e.g. GitHub). See the Nature Research guidelines for submitting code & software for further information.

Data

Policy information about availability of data

All manuscripts must include a data availability statement. This statement should provide the following information, where applicable:

- Accession codes, unique identifiers, or web links for publicly available datasets
- A list of figures that have associated raw data
- A description of any restrictions on data availability

All sequence data used for phylogenetic analyses is available from publicly available databases (fWormbase:Parasite (Release 12) 40 and ENSEMBL Metazoa (release 42)). Details of accession numbers of all sequences used for analysis are listed in the Supplementary Tables. All data used to determine the crystal structure has been deposited in the PDB (Accession number 6V4M). Any other data is available on request from the authors.

Field-specific reporting

Please select the one below that is the best fit for your research. If you are not sure, read the appropriate sections before making your selection.

☒ Life sciences ☐ Behavioural & social sciences ☐ Ecological, evolutionary & environmental sciences

For a reference copy of the document with all sections, see nature.com/documents/nr-reporting-summary-flat.pdf

Life sciences study design

All studies must disclose on these points even when the disclosure is negative.

Sample size	not applicable
Data exclusions	No data was excluded (except in rare cases where there were equipment failures during data acquisition such as sample blocking on FACS).
Replication	All experiments where replicates were required were performed three times with at least duplicate samples in each replicate.
Randomization	not applicable
Blinding	not applicable

Reporting for specific materials, systems and methods

We require information from authors about some types of materials, experimental systems and methods used in many studies. Here, indicate whether each material, system or method listed is relevant to your study. If you are not sure if a list item applies to your research, read the appropriate section before selecting a response.

Materials & experimental systems

n/a	Involved in the study	
☐	☒	Antibodies
☐	☒	Eukaryotic cell lines
☒	☐	Palaeontology and archaeology
☒	☐	Animals and other organisms
☒	☐	Human research participants
☒	☐	Clinical data
☒	☐	Dual use research of concern

Methods

n/a	Involved in the study	
☒	☐	ChIP-seq
☐	☒	Flow cytometry
☒	☐	MRI-based neuroimaging

Antibodies

Antibodies used	HA (Clone 12CA5, Roche), FLAG (Clone M2, Sigma-Aldrich), BCL-2 (Clone 7/BCL-2, BD Biosciences), beta-actin (Clone AC-74, Sigma-Aldrich), cytochrome c (Clone 7H8.2C12, BD Pharmingen).
Validation	no validation was performed other than that provided by manufacturers

Eukaryotic cell lines

Policy information about cell lines

Cell line source(s)	Wild-type and BAX-/-/BAK-/- cells were derived at the Walter and Eliza Hall Institute (WEHI)
Authentication	Cell lines were derived from mice in-house at WEHI. BAX-/-/BAK-/- cells were genotyped to confirm deletion. This was further confirmed by Western blotting.
Mycoplasma contamination	All cell lines have been tested in house and confirmed as mycoplasma negative using MycoALERT assays (Lonza)
Commonly misidentified lines (See ICLAC register)	not applicable

Flow Cytometry

Plots

Confirm that:

☒ The axis labels state the marker and fluorochrome used (e.g. CD4-FITC).

☒ The axis scales are clearly visible. Include numbers along axes only for bottom left plot of group (a 'group' is an analysis of identical markers).

☒ All plots are contour plots with outliers or pseudocolor plots.

☒ A numerical value for number of cells or percentage (with statistics) is provided.

Methodology

Sample preparation	Cells were seeded into 24-well plates and, 24 h later were treated with 50 µM Etoposide (Sigma-Aldrich) for 24 h. Both floating and adherent cells were then collected and washed with binding buffer (BD Bioscience) before staining them with Annexin V-APC (BD Biosciences) and propidium iodide (Sigma-Aldrich), and subsequently analysed by flow cytometery.
Instrument	Analysis - BD FACSCanto II, Sorting BD FACSAria II, BD Biosciences
Software	FLOWJO v10.5.3
Cell population abundance	Sorting was based on the presence of eGFP or mCherry transgenes introduced by lentiviral tranduction (co-expressed with our genes of interest). Transduction was generally highly efficient with >80% cells positive for eitehr transgene. Typically the top 30% of this population was used for cell-based experiments. Expression of genes of interest was confirmed by Western blotting (anti-FLAG or anti-HA).
Gating strategy	Cells were initially gated on FSC/SSC then further gated for live/dead populations using appropriate filter sets for APC (Annexin V) and propidium iodide. % Live cells were calculated as those that were negative for Annexin V and propidium iodide relative to those cultured in the presence of vehicle only.

☒ Tick this box to confirm that a figure exemplifying the gating strategy is provided in the Supplementary Information.
